# Distribution and Classification of Serine β-Lactamases in Brazilian Hospital Sewage and Other Environmental Metagenomes Deposited in Public Databases

**DOI:** 10.3389/fmicb.2016.01790

**Published:** 2016-11-15

**Authors:** Adriana M. Fróes, Fábio F. da Mota, Rafael R. C. Cuadrat, Alberto M. R. Dávila

**Affiliations:** Laboratório de Biologia Computacional e Sistemas, Instituto Oswaldo Cruz, FIOCRUZRio de Janeiro, Brazil

**Keywords:** serine β-lactamases, hospital sewage, metagenome, Hidden-Markov-Model, phylogenetic diversity

## Abstract

β-lactam is the most used antibiotic class in the clinical area and it acts on blocking the bacteria cell wall synthesis, causing cell death. However, some bacteria have evolved resistance to these antibiotics mainly due the production of enzymes known as β-lactamases. Hospital sewage is an important source of dispersion of multidrug-resistant bacteria in rivers and oceans. In this work, we used next-generation DNA sequencing to explore the diversity and dissemination of serine β-lactamases in two hospital sewage from Rio de Janeiro, Brazil (South Zone, SZ and North Zone, NZ), presenting different profiles, and to compare them with public environmental data available. Also, we propose a Hidden-Markov-Model approach to screen potential serine β-lactamases genes (in public environments samples and generated hospital sewage data), exploring its evolutionary relationships. Due to the high variability in β-lactamases, we used a position-specific scoring matrix search method (RPS-BLAST) against conserved domain database profiles (CDD, Pfam, and COG) followed by visual inspection to detect conserved motifs, to increase the reliability of the results and remove possible false positives. We were able to identify novel β-lactamases from Brazilian hospital sewage and to estimate relative abundance of its types. The highest relative abundance found in SZ was the Class A (50%), while Class D is predominant in NZ (55%). CfxA (65%) and ACC (47%) types were the most abundant genes detected in SZ, while in NZ the most frequent were OXA-10 (32%), CfxA (28%), ACC (21%), CEPA (20%), and FOX (19%). Phylogenetic analysis revealed β-lactamases from Brazilian hospital sewage grouped in the same clade and close to sequences belonging to Firmicutes and Bacteroidetes groups, but distant from potential β-lactamases screened from public environmental data, that grouped closer to β-lactamases of Proteobacteria. Our results demonstrated that HMM-based approach identified homologs of serine β-lactamases, indicating the specificity and high sensitivity of this approach in large datasets, contributing for the identification and classification of a large number of homologous genes, comprising possible new ones. Phylogenetic analysis revealed the potential reservoir of β-lactam resistance genes in the environment, contributing to understanding the evolution and dissemination of these genes.

## Introduction

The overuse and misuse of antibiotics in veterinary, farming, and human medicine are the main sources of antibiotic release in the environment and dispersion of multidrug-resistant bacteria worldwide, contributing to the evolution of drug resistance. However, antibiotics resistance determinants found in pathogens make up only a small fraction of the total Antibiotic Resistance Genes (ARGs) researched. It implies that the main reservoir for the ARGs may be non-pathogenic, in particular, those uncultured bacteria. This pool of ARGs is called environmental antibiotic resistome and hides an enormous molecular diversity (Wright, 2005; Henriques et al., 2006).

The knowledge on metagenome field (based on genomic analysis of microbial DNA from the environment) is increasing with the advances in high-throughput sequencing tools and bioinformatics software’s, revealing greater diversity and unexpected reservoirs of ARGs in the environment (Perry and Wright, 2013). Among them, there are β-lactamases (EC 3.5.2.6), which cleave the amine bond in the β-lactam ring of the antibiotic widely spread in Bacteria and Archaea domains (Petrosino et al., 1998).

In Gram-negative bacteria, β-lactamases are the main cause of resistance to β-lactam antibiotics and have been the subject of extensive microbiological, biochemical, and genetic investigations (Bush, 2001). They can be found in any kind of environment, including soil, sludge, water and human and animal microbiome (Henriques et al., 2006). β-lactamases present high levels of molecular diversity because they are usually encoded by genes that have been evolving rapidly and are generally present in mobile genetic elements as plasmids, phages, transposons, and also integrated into chromosomes (Petrosino et al., 1998; Paterson and Bonomo, 2005).

According to Ambler (Ambler, 1980), they are classified into four major molecular classes (A to D), based on amino acid sequence similarity. Class A (penicillinases), C (cephalosporinases), and D (oxacillinase) share structural homology but comprise evolutionarily distinct groups of enzymes, which have serine moiety as their active site, therefore classified as “Serine-β-lactamase” family (Paterson and Bonomo, 2005). On the other hand, Class B β-lactamases are analogous and contain zinc ions to disrupt the β-lactam ring (Livermore, 1995).

Serine β-lactamases share several highly conserved amino acid sequences with penicillin binding protein (PBPs), that is the target of β-lactams antibiotics forming a superfamily of penicillin-recognizing enzymes, together (Medeiros, 1997). These groups of enzymes include several variants (or types) with a distinct spectrum of activity, and new variants are constantly described around the world, including the wide spectrum activity β-lactamases (ESBL). Major classes of ESBLs include TEM, SHV, CTM-X (Class A) types and OXA type (Class D). They contain a number of mutations that allow them to hydrolyze expanded-spectrum β-lactam antibiotics.

Class A, C, and D β-lactamases differ, basically, by specific motifs (Medeiros, 1997; Matagne et al., 1999; Walther-Rasmussen and Høiby, 2007; Singh et al., 2009).

Although antibiotics resistance have been intensively studied in the clinical area, little is known about the diversity, distribution and environmental reservoir of these genes and their contribution to resistance in the clinical environment, in particular among the uncultured environmental bacteria (Allen et al., 2009b). Hospital sewage is a major source of dissemination of pathogens and other microorganisms into the aquatic environment and its subsequent return to the social environment. Therefore, this eﬄuent has a relevant role in the spread of resistance genes into the environment. Few studies have accomplished so far using shotgun metagenomics to estimate the presence of GRAs in different settings, including hospital sewage (Allen et al., 2009a; Kazimierczak et al., 2009; Donato et al., 2010; Torres-Cortés et al., 2011). Moreover, these studies have shown that this kind of methodology presents a great potential for discovering new genes, raising the number of metagenomic projects sequenced by NGS (Next Generation Sequencing) (Gill et al., 2006).

This work aims (a) to screen and classify homologs sequences of serine β-lactamases from classes A, C, and D present in different environments, including two Brazilian hospital sewages, (b) to analyze the phylogenetic relationship and diversity among β-lactamases classes screened, including some clinical strains, and (c) to classify and analyze diversity of serine β-lactamase types. Our results provided more information about differences in the conservation patterns along the putative β-lactamases sequences contributing to the current status of the knowledge concerning ARGs reservoir diversity.

## Materials and Methods

### Hospital Sewage Sampling and Processing

Sewage samples were collected from two public hospitals in the city of Rio de Janeiro, Brazil presenting different profiles. The first is located in the South zone (SZ) of Rio and is a community hospital. The second is located in the North zone (NZ) of the city and is a University-affiliated hospital. Both hospitals gave permission for the sampling by a declaration assigned by the directors. Samples from SZ hospital were obtained during the morning of April 27th of 2011, and from NZ hospital have been achieved on May 26th, in the same year. Samples were collected in polypropylene sterilized bottles and immediately transported to the laboratory on ice, until processing. Twenty liters of sewage was gathered from each hospital. Samples were filtered in membranes with different pore sizes [0.5 mm pore size (0.5 mm ø 47 ± 0.5 mm – Schleicher & Schuell), 1.2, 0.8, and 0.22 μm pore size (47 ± 0.5 mm diameter, Millipore^TM^)] using pump vacuum and then frozen at -80°C. Each membrane contained the cells from 100 mL of hospital sewage, approximately. One liter of each sample was used for physical-chemical analysis (pH, BOD_5_, QOD, phosphorous (P), and nitrogen (N)) were performed at DHES (Department of Health and Environmental Sanitation) located in ENSP/FIOCRUZ – RJ (Brazil). The results of this analysis are shown in Supplementary Table S1.

### DNA Extraction, Sequencing, and Quality Control

Cells on nine membranes (0.22 μm pore size), totalizing 900 mL of sewage each, were used to obtain 2 μg of metagenomic DNA from each hospital, using the Meta-G-Nome Isolation DNA kit for water (EPICENTRE Biotechnologies, Madison, WI, USA), according to manufacturer’s instructions. DNA integrity was determined by electrophoresis in a 1% agarose gel containing ethidium bromide (0.5 μg mL^-1^) and quantification and DNA quality analysis (260/280 nm) were performed using Nanodrop Spectrophotometer (ND 1000 V3.7, Nanodrop Technologies). DNA sequencing was generated by 454 pyrosequencing platform (Roche/454 GS FLX +), carried out in the National Laboratory of Scientific Computation, Petrópolis (LNCC), Brazil. Two runs were conducted, one for each sample. Samples collected from a hospital located in South Zone were named SZ and samples gathered from the hospital in North Zone were named NZ. Raw reads were trimmed for 454 inherent artifacts removal using CD-HIT-454 (version 4.5.4, default parameter, and 98% of identity option) (Beifang et al., 2010) and filtered by quality (Phred quality -Q = or > 20) and length (>100 bp) using Lucy program (version 1.2) (Chou and Holmes, 2001). Results of sequencing basic statistics are listed in **Table 1**. The metagenome sequencing data has been deposited at Genbank under the BioSample accession number SAMN05470414 and SAMN05473973.

**Table 1:** Statistics after sequencing and quality control steps of hospital sewage samples of South Zone (SZ) and North Zone (NZ) of Rio de Janeiro (Brazil).

Samples	SZ	NZ
N° of high-quality sequences	914,175	1,457,186
Lowest length (bp)	100	100
Highest length (bp)	1533	1775
Median	705	828
Mode	755	919

### Hospital Sewage Microbial Community

Taxonomic assignment of microbial community was assessed using BLASTX (Altschul et al., 1990) searches against NCBI NR database (13 Gb, 21,622,202 sequences on January 2013). BLAST output was analyzed by MEGAN4 software (“Metagenome Analyzer”) (Huson et al., 2007).

### Diversity Analysis of Serine β-Lactamases

#### Acquisition of Reference Protein Sequences to Create Hidden-Markov-Models Profiles

Full-length amino acid sequences of β-lactamases were obtained from ARDB^1^ (Antibiotic Resistance Genes Database) and UniProtKB^2^ and used as input for training sets. From the total β-lactamases sequences acquired, 69 belonged to Class A, 54 to Class C, and 72 to Class D. Multi-FASTA format files were generated for each class of enzyme. The presence of specific domains of β-lactamase class, including active-site motifs, was visually confirmed from the multiple alignments, using Jalview software (Waterhouse et al., 2009). A resumed pipeline is represented on Supplementary Figure S4.

#### Metagenomic Datasets

Two metagenomic databases were used: (i) CAMERA 2.0 (Sun et al., 2011) and (ii) IMG/M (Markowitz and Ivanova, 2006; Markowitz et al., 2008). Eighty-four metagenome datasets, available as nucleotide reads in FASTA format (∼126.4 GB), were downloaded from CAMERA 2.0 website (on May 2012). Those identical to IMG project were excluded. Protein sequences were obtained in FASTA format (∼20 GB) from 347 public environmental datasets available at IMG/M portal (on May 2012). Metagenomic datasets from CAMERA, available only as nucleotide sequences, were translated to amino acid sequences, in all six frames, using transeq program from the EMBOSS package (v6.1.0) (Rice et al., 2000). Environmental datasets included aquatic, wastewater, terrestrial, air, host-associated, solid waste, plant, and human microbiome projects.

#### Multiple Sequence Alignment

Multiple alignments were conducted using MAFFT (version 6.717b) (Katoh et al., 2002) using auto mode option. Jalview software v2.6.1 (Waterhouse et al., 2009) was used for visualizing and editing alignments, whenever mentioned.

#### Creation of Profiles and Screening for Candidate Serine β-Lactamases Sequences Using Hidden–Markov-Model

After multiple alignments of serine β-lactamases seeds (for A, C, and D classes), HMM profiles were created for each alignment using *hmmbuild* algorithm, from HMMER package (version 3.0) (Eddy, 2011). Profile HMMs (pHMMs) were graphically visualized using the LogoMat-M tool, available in Sanger Institute Web^3^, to check the amino acid conservation of pHMMs, including windows where principal motifs of serine β-lactamases were present. Searches were performed using pHMMs against public databases CAMERA 2.0, IMG/M, and also hospital sewage data. An inclusion *e*-value threshold of 10^-20^ was used for avoiding probable non-homologs sequences.

Sequences presenting less than 100 amino acids and redundant sequences were also removed. Logo-Mat of pHMM created for A, C, and D classes are showing in the Supplementary Figures S1–S3.

#### Annotation of Screened β-Lactamases Based on Conserved Domain Profiles

In order to provide additional evidence for annotation of putative serine β-lactamases sequences (obtained by HMMER searches) and reduce possible false positive results, position-specific scoring matrix (PSSM) searches against conserved domain profiles databases were performed by RPS-BLAST (v2.2.21) algorithm, with an inclusion of e-value threshold of 10^-5^. Conserved Domain Database (CDD, v2.25), Cluster of Orthologs Group (COG, v1.0) and Pfam (v24) were used as profiles databases. All three classes of β-lactamase were analyzed separately. Sequences presenting β-lactamases domains were used for further analysis.

### Phylogenetic Analysis of Putative β-Lactamases

Only almost-full-length metagenomic sequences of serine β-lactamases, which presented all specific motifs for each class of serine β-lactamase, and validated by RPS-BLAST, were used for the phylogenetic analysis. Amino acid sequences obtained from HMMER searches together with the reference sequences were grouped by class and aligned with MAFFT software (6.717b) with parameters “–maxiterate 1000 –localpair”. Outgroups were used for each β-lactamase class is described as follows: for Class A and D β-lactamases, Class C sequences were used, and for phylogenetic analysis of Class D β-lactamases, Class C sequences were used. Four sequences were used as the outgroup, within each β-lactamase Class (A, C, D). The nearest-neighbor sequences were used as a reference sequence, obtained by local alignment search (BLASTP – v 2.2.21) against RefSeq database (2780 Mb, March 2012). Functional annotation of the reference sequences used was confirmed by RPS-BLAST analysis against CDD (Conserved Domain Database) using a threshold of 10^-50^ for a stronger confidence. Amino acid sequences from β-lactamases diverge are very divergent, in particular between the isolates and non-cultivable. For this reason, poorly aligned positions and divergent regions were identified and removed using TrimAl (v.1.2) (Capella-Gutiérrez et al., 2009). The parameters used were “-gt 0.8 –st 0.001 –cons 60”. Phylogenetic analysis was carried out using RaxML-HPC (Randomized Axelerated Maximum Likelihood) v 7.4.2 (Stamatakis, 2006) with PROTGAMMA distribution and rapid bootstrapping (-x 12345) to estimate phylogeny using Maximum Likelihood (100 bootstraps replicates). Evolution model and gamma distribution parameters were determined by Modelgenerator (version 0.82) software (Keane et al., 2006). For phylogenetic analysis of Class A, an additional filter approach was used as too many metagenomic sequences (89,568) were retrieved after a search with HMM profiles against CAMERA (v2.0). Many sequences presented a high degree of similarity (>95%). Therefore, CD-HIT program was used to obtain sequences sharing the maximum similarity of 97% among them and removing high similar sequences. As the number of sequences was still high and Class A sequences of hospital sewage samples formed a cluster among them, two phylogenetic trees were reconstructed: one regarding Brazilian hospital sewage samples and another containing Class A from public databases.

### Classification of the Serine β-Lactamase Types

Serine β-lactamase sequences screened by pHMMs (created according to Section “Creation of Profiles and Screening for Candidate Serine β-Lactamases Sequences using Hidden-Markov-Model”), and had its function confirmed by the first RPS-BLAST analysis, were classified according to its subtype. For this propose, a new RPS-BLAST analysis was conducted against protein clusters, now using PSSM models of non-redundant and complete resistance genes as databases, on ARDB website^4^. An *e*-value threshold of 10^-5^ was used.

In order to check for the closest taxonomy of some major types classified, as KPC, BLASTP analysis was conducted against NCBI NR database (*e*-value threshold of 10^-5^). Relative abundance was calculated as the percentage of reads related to a specific β-lactamase type/total of reads of each β-lactamase class for each sample.

## Results

### Physical–Chemical Analysis and Sequencing

Physical–chemical analysis of pH, BOD_5_, QOD, phosphorous (P), and nitrogen (N) are shown in Supplementary Figure S1. The first sequencing run, from hospital sewage of SZ, yielded 914,175 reads (705 bp median read length). The second run sequencing, from hospital sewage of NZ, yielded 1,457,186 reads (828 bp median read length) (**Table 1**).

### Microbial Taxonomic Distribution of Brazilian Hospital Sewage

**FIGURE 1:**
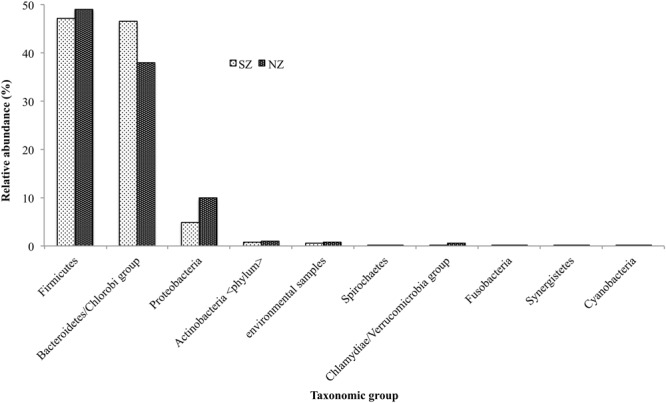
**Taxonomical distribution of the most predominant groups of Bacteria Phylum present in South Zone (SZ) and North Zone (NZ) samples from Rio de Janeiro (Brazil)**.

Bacteroidetes was the most prevalent phyla, accounting for 47 and 38% for SZ and NZ, respectively, and Firmicutes accounting for 47 and 49%, respectively. The second most abundant phylum was Proteobacteria, with 5 and 8%, for SZ and NZ samples, respectively. Actinobacteria, Spirochaetes, Chlamydiae, Fusobacteria, Synergistetes, and Cyanobacteria were among the seven others most abundant phyla, but all represented less than 1% for both samples (**Figure 1**).

### Search for Possible Serine β-Lactamase Genes

**FIGURE 2:**
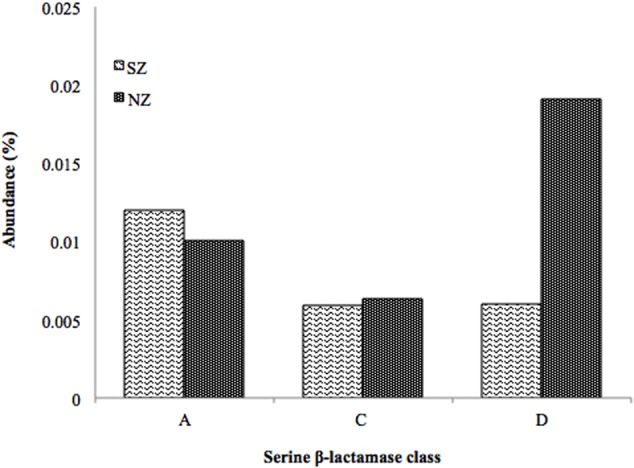
**Abundance (%) of serine β-lactamase classes **(A,C,D)** screened by the pHMM-based approach of SZ and NZ hospital sewage samples of Rio de Janeiro (Brazil)**. Abundance means the percentage of reads related to a specific β-lactamase class/total of reads of each metagenomes sample.

HMMER search for putative β-lactamases against public databases (CAMERA and IMG/M) retrieved 54,356 hits for Class A β-lactamases, 12,804 hits for Class C, and 2,698 hits for Class D. The search for putative β-lactamases in Brazilian hospital sewage metagenomes retrieved 242 reads for Class A (110 in SZ and 132 in NZ), corresponding to 0.012% and 0.010% abundance, 138 reads for Class C (54 in SZ and 84 in NZ,), corresponding to 0.006% (both samples) and 307 reads for Class D (55 in SZ and 252 in NZ), corresponding to 0.006 and 0.02% abundance, respectively (**Figure 2**).

Concerning public metagenomes results, RPS-BLAST confirmed 95 to 99% of the sequences retrieved for Class A and C, and 27 to 42% of the sequences retrieved as Class D β-lactamases. Furthermore, all reads scanned showed specific conserved motifs related to each β-lactamase class. Hospital sewage results revealed that more than 85% of the reads screened presented high similarity with conserved domains of serine β-lactamases, after RPS-BLAST. Only sequences presenting (i) the three principal β-lactamases motifs as the active site region (S–x–x–K for Class A and D, and S–x–S–K for Class C); S-D-N for Class A, Y-S (A)-N for Class C and Y-G (F)-N for Class D, and K-T-G triad; and (ii) 140-340 amino acids were used for phylogenetic analysis.

### Occurrence of Potential Serine β-Lactamases

#### Brazilian Hospital Sewage Metagenomes

Concerning serine β-lactamases screened from hospital sewage metagenomes, by the pHMMs-based approach, Class A presented 0.01% abundance for SZ sample, while C and D classes presented 0.006%. For NZ sample, Class D presented 0.02% abundance, Class A 0.01%, and Class C presented 0.006% abundance (**Figure 2**).

**FIGURE 3:**
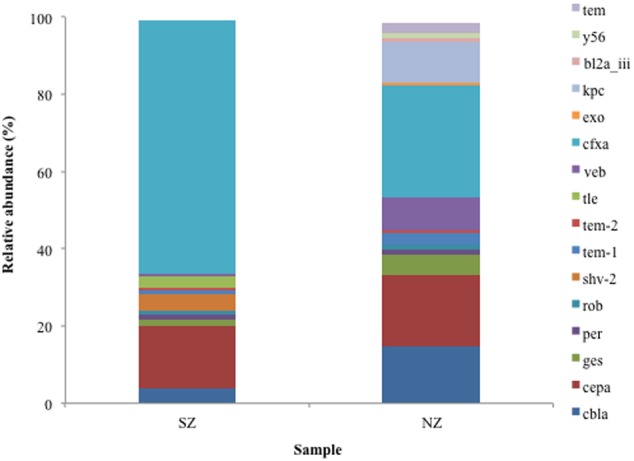
**Relative abundance (%) of Class A serine β-lactamases screened from SZ and NZ hospital sewage samples of Rio de Janeiro (Brazil).** Relative abundance means the percentage of reads related to each β-lactamase type or variant/total of Class A reads for each sample.

Analysis of serine β-lactamases Class A types revealed the CfxA type was frequent in both samples (SZ and NZ), presenting 65 and 29% of Class A reads, respectively. SZ sample presented 17% abundance for CEPA type and 5% for CBLA. Class A KPC type (*Klebsiella penumoniae* carbapenemase) was not detected in SZ sample but it was detected in NZ with a relative abundance of 11%. Taxonomic classification of KPC genes revealed closest relatives as *Klebsiella penumoniae, Pseudomonas aeruginosa* (KPC-2), *Serratia marcescens* (KPC-2), *Citrobacter freundii* (KPC-2), and *Escherichia coli* (data not shown). GES and VEB types were detected in both hospital samples (0.8 and 4.7% of GES in SZ and NZ samples, respectively, and 0.8 and 7.6% abundance of VEB in SZ and NZ, respectively) (**Figure 3**).

**FIGURE 4:**
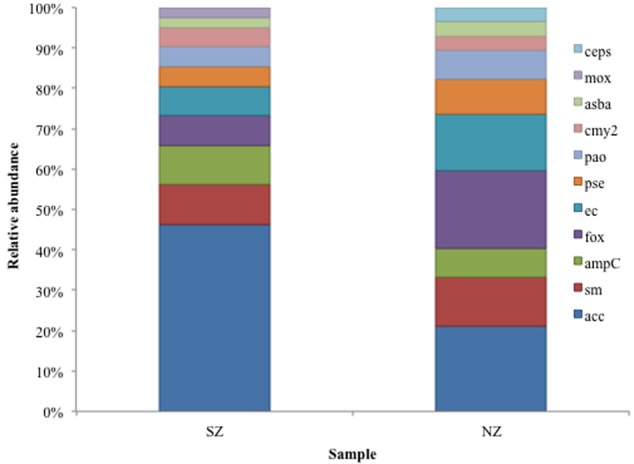
**Relative abundance (%) of Class C serine β-lactamases screened from SZ and NZ hospital sewage samples of Rio de Janeiro (Brazil).** Relative abundance (%), means the percentage of reads related to each β-lactamase type or variant/total of Class C reads for each sample.

**FIGURE 5:**
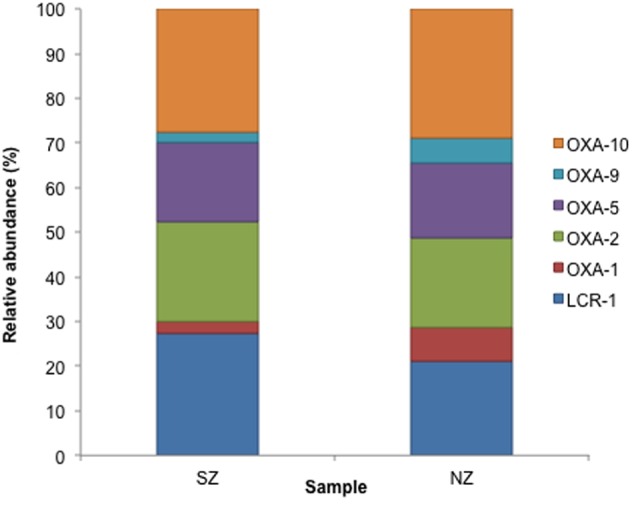
**Relative abundance (%) of Class D serine β-lactamases screened from SZ and NZ hospital sewage samples of Rio de Janeiro (Brazil).** Relative abundance means the percentage of reads related to each β-lactamase type or variant/total of Class D reads for each sample.

Class C ACC-type was detected in SZ sample (47% abundance) and Class C ACC and FOX types were detected in NZ sample, with 21 and 19% abundance, respectively (**Figure 4**). As regards to Class D, OXA-2, OXA-10 and LCR-1 types were detected in both samples, with relative abundances of 22 and 24% (OXA-2), 30 and 32% (OXA-10) and 27 and 20%, in SZ and NZ samples, respectively (**Figure 5**).

#### Public Metagenomes

Putative serine β-lactamases screened from public metagenomes databases revealed a higher abundance of Class A in projects as Acid Mine, Bison Metagenome, and Hydrothermal Vent, representing 0.3% (6137 sequences), 0.13% (3675 sequences), and 0.04% abundance (130 sequences), respectively. Class C β-lactamases were more abundant on DeepMed, Farm Soil, SAM, EPBRSludge, and PBSM projects, representing 0.02% (9 sequences), 0.01% (104 sequences), 0.001% (83 sequences), 0.007% (98 sequences), and 0.007% (2 sequences) abundance, respectively. Class D β-lactamases presented a higher abundance on Human Distal Gut, DeepMed, PBSM, and Whale Fall projects, with 0.006% (59 sequences), 0.004% (2 sequences), 0.003% (1 sequence) and 0.003% abundance (13 sequences), respectively (**Table 2**).

**Table 2:** Abundance (%) of Class A, C, and D serine β-lactamases screened by the pHMM-based approach, present on different metagenomes available on CAMERA public database.

Metagenome (CAMERA)	Total sequences	^∗^ β-lactamases	Abundance (%)	
CAM_-_PROJ_-_AcidMine	1914996	6137	0.32	**Class A**
CAM_-_PROJ_-_BisonMetagenome	2856498	3675	0.13	
CAM_-_PROJ_-_CAM_-_P0000101	4126902	2529	0.06	
CAM_-_PROJ_-_HydrothermalVent	297816	130	0.04	
CAM_-_PROJ_-_Sapelo2008	13091394	5483	0.04	
CAM_-_PROJ_-_BroadPhage	163607964	49147	0.03	
CAM_-_PROJ_-_CCMP1764	4688754	1307	0.03	
CAM_-_PROJ_-_WesternChannel	44128524	9963	0.02	
CAM_-_PROJ_-_HOT	34123506	4388	0.01	
CAM_-_PROJ_-_Yellowstone	179850	21	0.01	
CAM_-_PROJ_-_DeepMed	54288	9	0.017	**Class C**
CAM_-_PROJ_-_FarmSoil	830082	104	0.013	
CAM_-_PROJ_-_SAM	921042	83	0.009	
CAM_-_PROJ_-_EBPRSludge	1347096	98	0.007	
CAM_-_PROJ_-_PBSM	29886	2	0.007	
CAM_-_PROJ_-_WhaleFall	703956	38	0.005	
CAM_-_PROJ_-_HypersalineMat	774882	31	0.004	
CAM_-_PROJ_-_BisonMetagenome	2856498	94	0.003	
CAM_-_P_-_0000828	1167558	34	0.003	
CAM_-_PROJ_-_GutlessWorm	1882638	51	0.003	
CAM_-_PROJ_-_HumanDistalGut	936684	59	0.006	**Class D**
CAM_-_PROJ_-_DeepMed	54288	2	0.004	
CAM_-_PROJ_-_PBSM	29886	1	0.003	
CAM_-_PROJ_-_WhaleFall	703956	13	0.002	
CAM_-_PROJ_-_Yellowstone	179850	3	0.002	
CAM_-_PROJ_-_FarmSoil	830082	9	0.001	
CAM_-_P_-_0000523	3019674	25	0.001	
CAM_-_PROJ_-_CellCapture	3010068	22	0.001	
CAM_-_PROJ_-_GOS	76035108	517	0.001	
CAM_-_PROJ_-_HydrothermalVent	297816	2	0.001	

*Ten metagenomic projects presenting most abundant β-lactamases are presented.*

*^∗^Number of β-lactamases sequences screened by the pHMM-based approach and after removal of false positives by RPS-BLAST analysis.*

### Distribution of Serine β-Lactamases Based on Phylogenetic Analysis

#### Class A β-Lactamase in Brazilian Hospital Sewage

**FIGURE 6:**
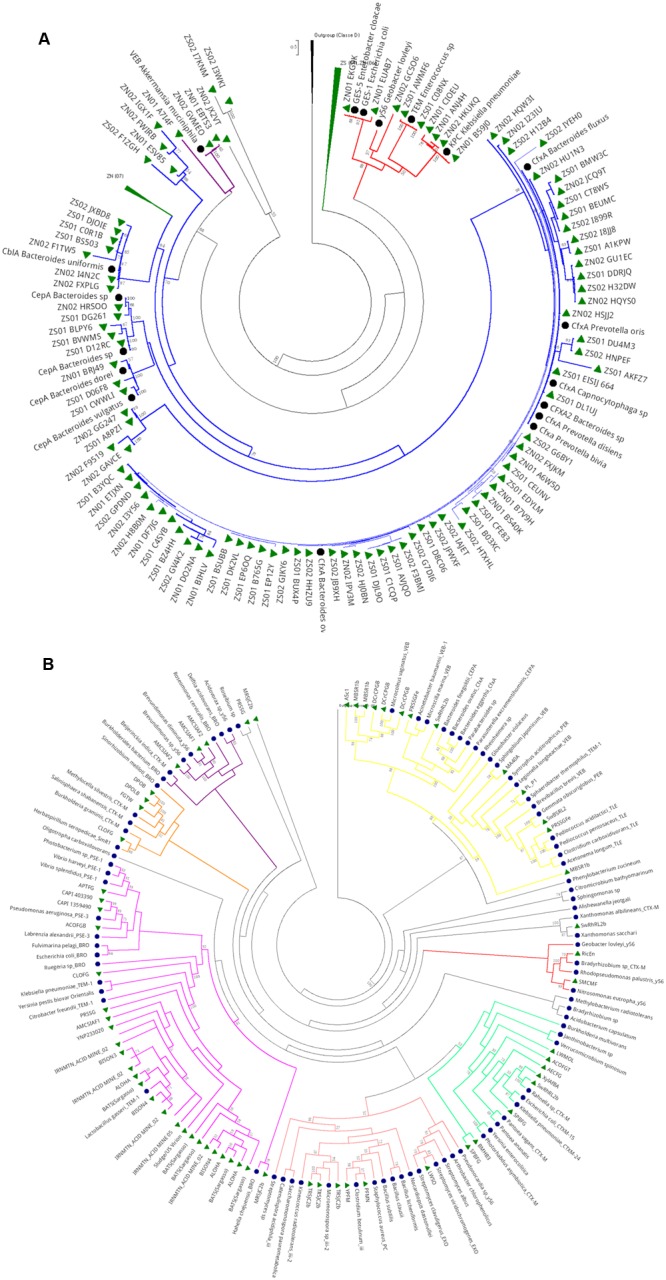
**Phylogeny of Class A β-lactamase retrieved from hospital sewage metagenomes of SZ and NZ of Rio de Janeiro (Brazil).** Representative β-lactamase sequences are indicated by a black closed circle indicates and hospital sewage sequences are indicated by a green closed triangle. Black closed triangle represents the outgroup (Class D) composed of many curated sequences collapsed. The phylogenetic tree was calculated by RAxML-HPC program, using Maximum Likelihood and WAG as the substitution model. The bootstrap value was 100 iterations were used and its values can be observed on the branches (above 50%). Scale bar indicates 0.5 amino acid substitutions per site. ZS stands for SZ (South Zone) and ZN stands for NZ (North Zone). **(B)** Phylogeny of Class A β-lactamase retrieved from public databases metagenomes (CAMERA and IMG/M). Representative β-lactamase sequences are indicated by a black closed circle and hospital sewage sequences are indicated by a green closed triangle. Black closed triangle represents the outgroup (Class D) composed of many curated sequences collapsed. The phylogenetic tree was calculated by RAxML-HPC program, using Maximum Likelihood and WAG as the substitution model. Bootstrap values (%), based on 100 iterations, can be observed on the branches (above 50%). Scale bar indicates 0.5 amino acid substitutions per site.

Phylogenetic analysis of Class A genes from the hospital sewage revealed three main clades (**Figure 6A**). The first clade (branches in red) is composed of sequences from the two hospital samples (SZ and NZ) and grouped close to distinct Class A β-lactamase types, which were associated with representative sequences of GES-1, TEM, and KPC types. The second clade (branches colored in blue), which comprised the majority of the tree, presented a shorter phylogenetic distance between the sequences (short branches). This clade is composed of sequences from both hospital sewage and grouped close to Class A genes from Bacteroidetes phylum as *Prevotella, Capnocytophaga* and *Bacteroides* and different β-lactamase types as CfxA, CepA, and CblA. The third clade (branches in purple) is composed of sequences from both hospitals, which grouped closer to a gene characterized as Class A β-lactamase VEB type of *Akkermansia muciniphila* (Verrucomicrobia).

#### Class A β-Lactamase in Public Metagenomes

Regarding the phylogenetic analysis of Class A serine β-lactamase screened from environmental data from public databases (**Figure 6B**), seven predominant clades were observed. Most of these sequences clustered close to Firmicutes and Proteobacteria groups. On the first clade (colored in yellow) three subclades were formed mainly by different terrestrial (MBSR1b, DcrCPGB, A5c1) projects. The first subclade clustered close to a representative Class A β-lactamase from *Microcoleus vaginatus* (Cyanobacteria), while the second clustered together with Class A β-lactamases, also from terrestrial environments (SwRhRL2b e PRSSGFe) but grouped close to β-lactamases sequences from Proteobacteria and Bacteroidetes. The third subclade was formed by Class A sequences of distinct phyla as Proteobacteria, Chloroflexi, Planctomycetes. and Firmicutes, from distinct environments as terrestrial (PL_P1, SwRhRL2b e PRSSGFe) and projected (MA40A). The vast majority of the environmental sequences of β-lactamases belonging to Class A were highly divergent from curated β-lactamases, “most from clinical origin”. This result shows how environmental Class A β-lactamases diverge from the clinical in an evolutionary way. Clade 2 (colored in red) was formed by sequences from soil environment (RicEn and SMCMF), which grouped close to CTX-M and y56 from *Bradyrhizobium* sp. and *Nitrosomonas eutropha.* Another big cluster (colored in pink) was formed with public databases sequences, from projects as Sludge (Australia and the USA), Human Gut, Bison Metagenome, Acid Mine, and Yellowstone Hot Springs, Singapore indoor air filters, Hydrothermal Vent, Lake Washington. They grouped closer to two SHV (5 and 71) of *Acinetobacter baumanii* and *Shigella* sp., indicating a probable classification of SHV variants of this cluster.

#### Class C β-Lactamase in Sewage Hospital and Public Metagenomes

**FIGURE 7:**
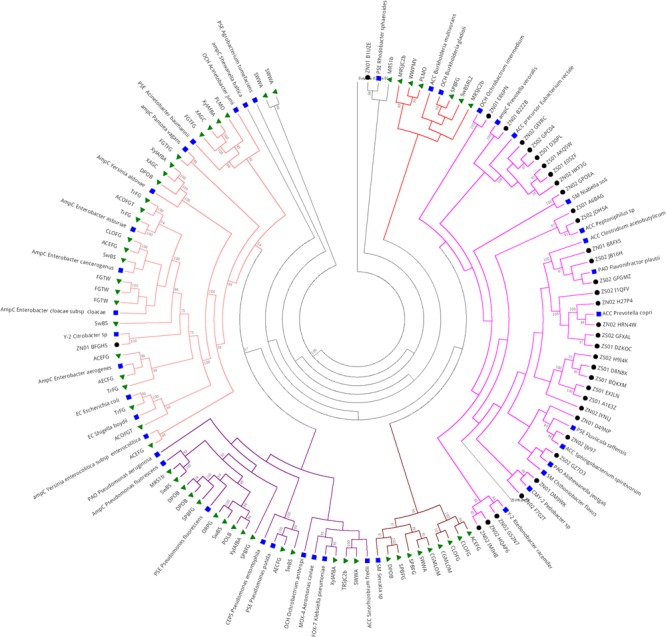
**Phylogeny of Class C β-lactamase retrieved from hospital sewage metagenomes of SZ and NZ of Rio de Janeiro (Brazil), and from public databases (CAMERA/IMG/M).** Representative β-lactamase sequences obtained from RefSeq database are indicated by a blue closed square, sequences from public databases are indicated by a closed green triangle, and hospital sewage sequences of Rio de Janeiro are indicated by a black closed circle. Black closed triangle represents the outgroup (Class D) composed of many curated sequences collapsed. The phylogenetic tree was calculated by RAxML-HPC program, using Maximum Likelihood and WAG as the substitution model. Bootstrap values (%), based on 100 iterations, can be observed on the branches (above 50%). Scale bar indicates 0.5 amino acid substitutions per site. ZS stands for SZ (South Zone) and ZN stands for NZ (North Zone).

Metagenomic data public databases clustered with sequences classified as Class C β-lactamase, most of ACC type (**Figure 7**). Short branches among the sequences from the same clade suggest a close relationship and a brief evolutionary distance. Most of the Class C sequences retrieved from the public databases that clustered together were from projects related to insect microbiome, usually associated with symbiotic bacteria (74%, 46 sequences). Just 6.4% of the sequences grouped with others sequences from sludge’s projects (4 sequences) and 19.3% were from soil projects. Five main clades were formed, with distinct clades formed by ACC and OCH types from *Burkholderia multivorans* and *B. gladioli* (for clade 1) and ACC, PAO, PSE, OCH, SM, CMY-2, Y-2, and AmpC types associated to Bacteroidetes and Firmicutes in clade 2, which constituted mostly sequences from Brazilian hospital sewage (SZ and NZ) and representatives sequences. Clades 3 and 4 (colored in green and purple, respectively) were formed by sequences from distinct metagenomes projects as interaction of symbiotic fungi and arthropods (mostly beetle), soil and deep water, close to SM subtype from *Serratia* sp., and ACC, OCH, PSE, CEPS, ampC, PAO, MOX-4 (ESBL), and FOX-7, most of them belonging to Proteobacteria phyla. The fifth clade (branches in salmon) was clustered with sequences of distinct projects, including sewage, soil, and microbe interaction with arthropods as ants, beetles, and termites, which clustered close to OCH, AmpC, PSE, Y-2, and EC types, and most of them also belonged to Proteobacteria phyla (**Figure 7**).

#### Class D β-Lactamase in Sewage Hospital and Public Metagenomes

**FIGURE 8:**
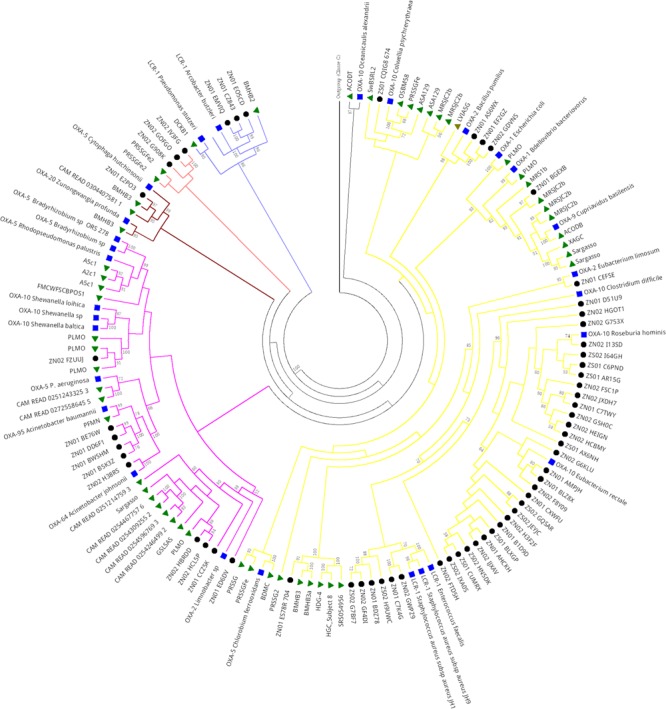
**Phylogeny of Class D β-lactamase retrieved from hospital sewage metagenomes of SZ and NZ of Rio de Janeiro (Brazil), and from public databases (CAMERA/IMG/M).** Representative β-lactamases sequences obtained from RefSeq database are indicated by a blue closed square, public databases sequences are indicated by a closed green triangle, and hospital sewage sequences of Rio de Janeiro are indicated by a black closed circle. Black closed triangle represents the outgroup (Class C) composed of many curated sequences collapsed. The phylogenetic tree was calculated by RAxML-HPC program, using Maximum Likelihood and WAG as the substitution model. Bootstrap values (%), based on 100 iterations, can be observed on the branches (above 50%). Scale bar indicates 0.5 amino acid substitutions per site. ZS stands for SZ (South Zone) and ZN stands for NZ (North Zone).

Phylogenetic analysis of Class D serine β-lactamase of public metagenomes revealed five main clades (**Figure 8**). The first clade (branches in purple) was formed by different projects including soil, freshwater, marine, sewage treatment plant, fungi and arthropod interaction, extreme environments, and also human gut microbiome and Brazilian hospital sewage (NZ and SZ). Despite this, most of the Brazilian hospital sewage sequences clustered together, in subclade, apart from the public metagenomes sequences. SZ and NZ sequences grouped close to Class D OXA-10 type of *Eubacterium rectale* and *Roseburia hominis*, which compose the human gut microbiome. In clade 1, most of the metagenomic sequences of public databases grouped close to OXA-1, OXA-2, OXA-5, and OXA-9 types and hospital sewage sequences (SZ and NZ) grouped close to OXA-10 and LCR-1.

Clade 2 (colored in pink) also presented a similar pattern to clade 1 as was formed by a cluster of Class D genes of distinct environments, including Brazilian hospital sewage (SZ and NZ), but this sequences grouped closer to OXA-10, OXA-64, OXA-95, and OXA-5 types.

Clades 3 and 4 presented similar patterns. Clade 3 was nested by sequences surrounding OXA-20 and OXA-5 types, and, in the second one, sequences grouped closer to LCR-1. Sequences from Brazilian hospital sewage grouped closer to Class D sequence of a bacteria belonging to Firmicutes group, while public metagenomic sequences grouped closer to Class D sequence of Proteobacteria group (**Figure 8**).

## Discussion

### Brazilian Hospital Sewage Metagenomes

#### Microbiome Profile

Hospital sewage composition is variable and related to the main clinical activities that each hospital develops. Usually, it is composed of human waste, representing a supportive environment to the development of diseases and containing a high number of pathogenic and antibiotic resistant microorganisms (Ribeiro, 2005). The human gut microbiome comprises all the three domain of life (Bacteria, Archea, and Eukarya), and also viruses, but bacteria are the main domains present (Bäckhed et al., 2005). Qin et al. (2010) defined the gut microbiome core, which included Bacteroidetes (genus Bacteroides as the most abundant) and Firmicutes (*Clostridium, Ruminococcus*, and *Eubacterium* being the most abundant) as the prevalent groups (Eckburg et al., 2005; Gill et al., 2006). Together, they represent more than 90% of the human gut microbiome (Eckburg et al., 2005) and also prevail on human feces. Regarding the Brazilian hospital sewage, a similar pattern was found in both samples (SZ and NZ), with Firmicutes being the most abundant group (40–50%) (**Figure 1**).

On the other hand, Synergistetes and Cyanobacteria were among the top 10 most abundant phyla on hospital sewage samples, but in very low abundance (0.2 and 0.06% for SZ and NZ, respectively). Synergistetes are anaerobic Gram-negative, rod-shaped bacteria present in human animal, terrestrial and ocean habitats (Vartoukian et al., 2007) including wastewater treatment plant (Ganesan et al., 2008). On the other hand, Cyanobacteria group is not considered part of the human gut microbiome, but it is very common in freshwater that eventually may end up on the sewage. Regarding the prevalent genera found and belonging to Firmicutes and Bacteroidetes, those are in accordance with the literature describing the composition of human gut microbiome (Palmer et al., 2007; Mariat et al., 2009): *Bacteroides, Eubacterium, Blautia, Clostridium, Ruminococcus, Faecalibacterium*, and *Coprococcus* are among the major genus found, suggesting the main composition of SZ and NZ hospital sewage samples is human feces, as expected.

#### Abundance of Serine β-Lactamase Classes

Both hospitals presented similar patterns of Class A and C β-lactamases, but with different percentage values (Class A was more abundant), and Class D presented the highest abundance of all three classes, in NZ (0.02%) sample (**Figure 2**). The difference observed could be due to the antibiotic used in each hospital and their distinct profiles. NZ is a University-affiliated hospital, which treats chronic diseases and has more clinical departments, and SZ is a community hospital, including emergencies and traumas. Also, Rio de Janeiro is a big city with highly different features, including social-economical, hygiene and sanitary conditions, and the NZ is characterized by presenting a lower social-economical, hygiene, and sanitary conditions than SZ.

Comparison of our Brazilian hospital sewage data with public environmental metagenome databases, showed that hospital sewage metagenomes present the highest percentage values of Class C serine β-lactamase (0.006% in both samples) and Class D (0.006% in SZ and 0.02% in NZ). Interestingly, others sewage metagenomes present in public databases did not appear on the top 10 projects with most abundant serine β-lactamases.

#### Relative Abundance and Phylogenetic Analysis

Inference of the relationship among serine β-lactamases classes is tough, as they were diverging much time ago, with several horizontal transfer events occurring. Therefore, they can share a poor similarity, even between sequences of the same class (less than 30% sequence identity) (Hall and Barlow, 2004; Lee et al., 2016). So, it is important to use sensitive detection methodologies to search for more remote homologs. In general, most of the protein families exhibit conservation patterns along the amino acid sequences, in multiple alignments, by forming blocks. Such patterns are the result of evolutionary pressure to maintain structures and proteins function. Some previous studies of metagenomes involving mining of target genes as chitinases (Romão-Dumaresq et al., 2014), PKS and NRPS (Cuadrat et al., 2016) used HMM profiles obtaining good results and sensitivity. Our HMM profiles for serine β-lactamases, followed by our RPS-BLAST analysis, revealed most of the retrieved metagenomic sequences belongs to the β-lactamase family, confirming the efficacy of profile approaches for this propose.

Class A is the largest class (in numbers) of β-lactamase, and more than 45 types have already been determined, so far. Nevertheless, their catalytic properties and primary sequences differ considerably, making them a highly diverse class (Matagne et al., 1999). By phylogenetic analysis, Regarding Brazilian hospital sewage samples, SZ and NZ, CfxA (65%, 28.5%) and CEPA (17%, 20%) types were more abundant in Class A, respectively. According to previous works (Tribble et al., 1997; Ferreira et al., 2007), horizontal gene transfer of β-lactamase is frequent between Bacteroides species, including the *cfxA* gene, usually associated to a conjugative transposon (Tn4555). Its presence can result in high levels of cefotoxin (Tribble et al., 1997). The great abundance of CfxA can be related to the high diversity of Bacteroides found in hospital sewage samples, suggesting the dissemination of this gene among Bacteroides species from human gut and indicating a possible reservoir of β-lactams resistance genes, especially cephalosporins. Another Class A β-lactamase, the KPC (*K. pneumonieae* carbapenemase) type, was also found in high relative abundance in NZ hospital sewage (11% of Class A reads), belonging to different bacteria genus. The high abundance of KPC in NZ hospital sewage sample suggests a potential source of transmission of carbapenemases to others environmental bacteria, including the human gut. The presence of KPC was also reported in Guanabara Bay and Flamengo Beach, in Rio, close to where some sports as sailing are practiced, including Olympic competitions of 2016 (Fistarol et al., 2015; Montezzi et al., 2015). Other ESBLs families as PER, VEB, and GES are not commonly described, just in France and Turkey (PER) (Vahaboglu et al., 1995; De Champs et al., 2002) France and South Africa (GES) (Dubois et al., 2002; Nordmann and Poirel, 2002), and Thailand, France, Kuwait, India, and China (VEB) (Girlich et al., 2001; Aubert et al., 2004; Jiang et al., 2005; Naas et al., 2006). They are usually found in *Pseudomonas aeruginosa* strains and they were also reported in *Acinetobacter baumannii*. In the present work, we detected these three families using bioinformatics tools, and the presence of GES subtype is worrying as they are capable of hydrolyzing carbapenems and are plasmid-encoded (Bush and Jacoby, 2010), being a public health concern as they can be exchanged with other species. Up to now, tiny little information is available about these genes in Brazil (Silva and Lincopan, 2012). Nevertheless, the presence and dispersion of GES in the bacterial community are a worrying concern for public health.

AmpC β-lactamases are usually found on the chromosomes of Gram-negative bacteria, but they have been detected also on plasmids that code ESBLs (Philippon et al., 2002), and, frequently carry other antibiotic resistance genes as chloramphenicol, aminoglycosides, sulfonamides, tetracycline, trimethoprim and even mercury (Papanicolaou et al., 1990; Bauernfeind et al., 1996, 1997; Bradford et al., 1997; Stapleton et al., 1999). They also confer resistance to 7-α-methoxy-cephalosporines and are not inhibited by the available commercial β-lactamases inhibitors, representing a new threat to health. Furthermore, strains presenting loss of porines on the external membrane can be resistant to carbapenems (Philippon et al., 2002). CMY-type enzymes are the most frequent reported plasmid-mediated AmpC β-lactamase (Jacoby, 2009), but in Brazilian sewage samples CMY-2 type were detected in lower abundance compared to ACC, FOX, and SM types (**Figure 8**). ACC (Ambler Class C) is a new type of AmpC, detected on chromosomes, and ACC-1 was the first kind of AmpC found on a plasmid, in *K. pneumonieae* and share low identity (<50%) with AmpC of the pathogens tested (Bauernfeind et al., 1999). The presence of ACC is very worrying as they are not inhibited by clavulanic acid and reports about its detection have been raising in the last decade, in clinical isolates of all over the world (Girlich et al., 2000; Nadjar et al., 2000; Makanera et al., 2003; Bidet et al., 2005; Miró et al., 2005; Ohana et al., 2005). In Brazil, the presence of isolates carrying plasmid-mediated AmpC has been sporadically reported and is still scarce (Castanheira et al., 2007; Pavez et al., 2008; Campana et al., 2013). However, in hospital sewage of Rio de Janeiro, they were one of the most abundant Class C types (5–8% of Class C reads).

OXA type (Class D) β-lactamases are characterized by a high hydrolytic activity against oxacillin. Amino acids mutations on these enzymes can also confer extended-spectrum resistance to (ESBL) and low susceptibility to clavulanic acid (Bush et al., 1995). On the present work, only 20% of the probable Class D had their function checked by RPS-BLAST analysis against conserved domains databases. Class D types present a low similarity among them (∼20% identity) as they were classified in this category because they introduced the same activity (on oxacillin) and had related substrates, but not by their primary structure (Jacoby, 2006). Hospital sewage sample NZ presented a higher abundance of Class D, and phylogenetic analysis revealed that these enzymes grouped close to Firmicutes or *Roseburia* genus, which includes commensal strains in the human gut. None of the types classified are considered ESBLs.

Our study showed the presence of a high diversity of serine β-lactamases types present on Brazilian hospital sewage, indicating a possible dispersion to other environments, as the rivers and ocean, if not properly treated before disposal.

### Public Metagenomes Present Very Distinct Serine β-Lactamases Profiles

Environmental public databases as CAMERA and IMG/M were used to explore the diversity and distribution of serine β-lactamases on the environment. Also, we intended to look for the phylogenetic relationship among serine β-lactamases present on the environment and clinical pathogens. Class A, C, and D serine β-lactamases obtained from public repositories presented very distinct patterns between the environments. Class A CTX-M and Class A TEM-type were more abundant on public environmental databases, including projects with a less direct anthropic impact as Sargasso Sea and Yellowstone Nymph lake metagenomes.

Yellowstone Bison Hot Spring (sites 3 and 4) and Acid Mine presented a higher abundance of Class A, which also was corroborated by phylogenetic analysis (**Figure 6B**) and sequences were grouped in a bigger cluster (colored in pink). Interestingly, these two environments are composed of extreme conditions as high temperature (>68°C) (Swingley et al., 2012) and low pH (acidic), respectively. On the other hand, the abundance of Class C and D were very low compared to Class A.

Class C was prevalent on very distinct projects concerning soil (Farm Soil) and aquatic environment (DeepMed). Farm Soil project is related to microbial community of the surface of an agriculture farm soil (0–10 cm) located in Minnesota (EUA) (Tringe et al., 2005) They are known as a reservoir of antibiotic genes as usually have a high concentration of antibiotics used to treat the animals, and their use is correlated with the rise and spread of associated resistance genes in human pathogenic bacteria, as well as the direct transfer of antibiotic-resistant bacteria from animals to humans (Levy and Marshall, 2004; Smillie et al., 2011; Forsberg et al., 2012; Zhu et al., 2013). Surprisingly, the second public project was DeepMed metagenome from a deep region of the Mediterranean Sea, also known as batpelagic zone (1000–4000 m of depth), where the sunlight does not reach and the temperature is very low and pressure is high (Martín-Cuadrado et al., 2007).

Regarding Class D β-lactamases, they were more abundant on very distinct environments as on Human Distal Gut, PSBM (Microbial community from sand beaches of Pacific) and also DeepMed projects. Human Distal Gut has been reported about a vast diversity of antibiotic resistance genes, including β-lactamases. This result suggests heterogeneity of β-lactamase classes between different locations and ecosystems and maybe a dispersion of this enzymes between the various regions even in places that should not have high concentrations of antibiotics. Studies about mobilome may help to confirm and connect this information about the dispersion of β-lactamases by mobile genetic elements. Our pHMMs were able to retrieve sequences with probable β-lactamase activity, initially described as “hypothetical protein” on the public databases used (IMG/M and CAMERA), after the functional inference using RPS-BLAST analysis and phylogeny.

## Conclusion

Although antibiotic resistance represents a major public health concern, this phenomenon has been largely overlooked in environmental settings.

The presence of the active site and specific motifs from each β-lactamase class in most of the scanned sequences from environmental datasets supports functional annotation, improved by the pHMM-based approach. Multiple sequence alignments of the β-lactamase domain revealed conserved regions corresponding to structural elements required for the serine protease active center formation, corroborating with functional annotation of serine β-lactamase groups. Phylogenetic analysis classified β-lactamases from public and hospital sewage of Rio de Janeiro metagenomes, revealing a high diversity of these resistance genes in the environment, as its dissemination. Diversity patterns found within the β-lactamase gene families studied do not correspond to those reported in studies on characterized clinical isolates, not even to Brazilian hospital sewage. They are evolutionarily distant from the clinic, especially those detected in the Proteobacteria group. The two-hospital sewage samples of Rio presented genes of all the three classes of serine β-lactamases (A, C, and D), including ESBLs and carbapenemase, suggesting the circulation of these genes on both hospitals, including not common ESBLs as PER, VEB, and GES types. Also, the present data contributed to the description, spread, and diversification of AmpC-plasmid encoded types.

Therefore, new data and results generated in this study will help a better understanding of the diversity and evolution of serine β-lactamases, providing favorable settings for the horizontal transfer of mobile genetic elements encoding antibiotic resistance genes. Understanding sources and mechanisms of antibiotic resistance are critical for developing effective strategies for reducing their impact on the public and environmental health, and also contributes to the establishment of novel genes links between the environment and the clinic. These findings also indicate an alert to the city of Rio de Janeiro because they are relevant sources of contamination of rivers and beaches that surround the city.

## Author Contributions

AF and AD were the principal authors and were involved in all steps of this work, and FM, RC were equally involved and made substantial and intellectual contribution to the work.

## Conflict of Interest Statement

The authors declare that the research was conducted in the absence of any commercial or financial relationships that could be construed as a potential conflict of interest.

## Footnotes

1 http://ardb.cbcb.umd.edu/

2 http://www.ebi.ac.uk/uniprot/

3 http://www.sanger.ac.uk/resources/software/logomat-m/

4 http://ardb.cbcb.umd.edu
