# Supplementary material for: Distribution and Classification of Serine β-Lactamases in Brazilian Hospital Sewage and Other Environmental Metagenomes Deposited in Public Databases
Source: Front Microbiol. 2016 Nov 15;7:1790. doi: 10.3389/fmicb.2016.01790 (PMC5108929; doi:10.3389/fmicb.2016.01790)
Supplement: Supplementary file 1 [file Data_Sheet_1.DOC]

Supplementary Material

**Distribution and classification of** **Serine β-lactamases in Brazilian Hospital Sewage and Other Environmental Metagenomes deposited in Public Databases**

**Adriana M. Fróes^1^, Fábio F. da Mota^1^, Rafael R. Cuadrat^1^, Alberto M.R. Dávila^1*^* Correspondence:** Alberto M.R. Dávila: davila@fiocruz.br

# Supplementary Data

**1.1. Initials and description of metagenomic projects used for phylogenetic analysis, from CAMERA and IMG/M public databases**

**Environmental projects:**

**Aquatic**

**GSLSAS:** Saline water microbial communities from Great Salt Lake Utah sample from South Arm Stromatolite.

**LVIA5G:** Freshwater microbial communities from Lake Vostok at Ice accretion.

**ASA129:** Marine sediment archaeal communities from Santa Barbara Basin CA that are methaneoxidizing sample 912 cm ANME Sed A12 912 cm.

**OSBM58:** Marine microbial communities from Deep water Horizon Oil Spill sample BP Oil Spill BM58.

**GSLSAS:** Saline water microbial communities from Great Salt Lake Utah sample from South Arm Stromatolite South.

**FMCWFSCBPOS1:** Fossil microbial community from Whale Fall at Santa Cruz Basin of the Pacific Ocean Sample *1.

**YNP233020:** Hot spring microbial community from Yellowstone Hot Springs sample from Nymph Lake site NL10.

**LWMOL:** Methylotrophic community from Lake Washington sediment Methanol enrichment**.**

**BISON3:** Yellowstone Bison Hot Spring Pool 3 Metagenome (CAM_PROJ_Bison_Metagenome).

**BISON4:** Yellowstone Bison Hot Spring Pool 4 Metagenome (CAM_PROJ_Bison_Metagenome).

**IRNMTN_4:** Acid Mine, Richmond Mine, Iron Mountain (EUA), site 4 (IRNMTN_4).

**IRNMTN_5:** Acid Mine Richmond Mine, Iron Mountain (EUA), site 5, temperature 38ºC (IRNMTN_5).

**ALOHA:** Marine planktonic communities from Hawaii Ocean Times Series Station (CAM_PROJ_HOT/ALOHA).

**BATS (Sargasso)**: Sargasso Sea Planktonic Microbial Community - Metagenome isolated from the BATS station (Bermuda Time Series Station) in the Sargasso Sea (CAM_PROJ_BATS).

**Terrestrial**

**MRS1b:** Miscanthus rhizosphere microbial communities from Kellogg Biological Station MSU sample Replicate 1.

**MRSJC2b:** Mesophilic rice straw.

**TRSJC2b:** Thermophilic rice straw.

**SwBSRL2:** Switchgrass rhizosphere microbial community from Michigan US sample from Rose Lake bulk soil RL2.

**FACENCTA:** Soil microbial communities from sample at FACE Site 4 Nevada Test Site Crust CO2.

**DcrCPGB:** Soil microbial communities sample from Dark Crust Colorado Plateau Green Butt.

**MBSR1b:** Miscanthus rhizosphere microbial communities from Kellogg Biological Station MSU.

**LWSO:** Sediment microbial communities from Lake Washington Seattle for Methane and Nitrogen Cycles original sample replicate 1.

**LWMOL:** Methylotrophic community from Lake Washington sediment Methanol enrichment.

**SMCMF:** Soil microbial communities from Minnesota Farm.

**PRSSG:** Feedstockadapted consortia SG only.

**PRSSGFe:** Feedstockadapted consortia SG + Fe.

**A5c1:** Active Layer.

**PL_P1:** Permafrost Layer P1.

**TRSJC2b:** TOT sample at FACE Site 4 Nevada Test Site Crust CO2.

**RicEn:** Endophytic microbiome from Rice.

**Microbial-host interaction**

**XAGC:** *Xyleborus affinis* microbiome from Bern Switzerland sample of gallery community.

**XylAfBA:** *Xyleborus affinis* microbiome from Bern Switzerland sample of adult community - Ambrosia beetle adult.

**APTFG-** Fungus garden microbial communities from *Apterostigma dentigerum*.

**ACODB:** Fungus garden microbial communities from *Atta colombica* in Panama sample from dump bottom.

**ACODT:** Fungus garden microbial communities from *Atta colombica* in Panama sample from dump top.

**ACOFGB:** “Fungus garden microbial communities from *Atta colombica* in Panama- sample from fungus garden bottom”

**ACOFGT:** Fungus garden microbial communities from *Atta colombica* in Panama-sample from fungus garden top.

**AECFG:** Fungus garden combined.

**DPOB:** Mountain Pine Beetle microbial communities from Grand Prairie Alberta sample from Hybrid pine.

**DPOLB:** Mountain Pine Beetle microbial communities from McBride British Columbia Canada sample from Lodgepole pine.

**FGTW:** Fungus growing Termite worker microbial community from South Africa sample from Oerlemans Farm.

**CLOFG:** *Cyphomyrmex longiscapus* fungus garden.

**SPBFG:** *Dendroctonus frontalis* Fungal community.

**HDG:** Human Distal Gut.

**HGC:** Human Gut Community.

**Engineered**

**PLMO:** Wastewater treatment plant plasmid pool from Canton de Vaud Switzerland sample from Preverenges Morges.

**BMHB3:** Biofuel Metagenome.

**MA40A:** Mixed alcohol bioreactor microbial communities from Texas AM University sample 40ºC degree reactor.

**Sludge/US_Virion –** sludge from USA.

**SRS054956:** Hypothetical protein Human Stool microbiome from visit number 1 of subject 737052003.

**CAPI:** Wastewater treatment Type I Accumulibacter community from EBPR Bioreactor.

**PFMN:** Fecal microbiome of Swine from Yorkshire Ohio.

**Air**

**AMCSIAF1:** Air microbial communities Singapore indoors air filter 1.

**AMCSIAF2:** Air microbial communities Singapore indoors air filter 2.

# Supplementary Figures and Tables

## Supplementary Figures

**HMM profile of serine β-lactamases**

# Figures S1, S2 and S3 show the logo with the main conserved regions of HMM profiles built for each class of β-lactamases. In the logo of class A, window one (1) is presented the active site Ser-X-X-Lys along with the amino acid Phe (F) which precedes the catalytic site (on position 43) forming a conserved region typical found in class A β-lactamases. The amino acids composing the catalytic site, between Ser and Lys vary greatly and they were: (i) in the first position were Ser (Serine), T (Threonine) and V (Valine); (ii) in the second site, after Ser, Phe (F) presented as more frequent amino acid. The second conserved motif was Ser-Asp-Asn (S-D-N), which appeared with a higher frequency, including the amino acid A in the position 111. Amino acids composing the third conserved motif, (Figure S1) were the triad Lys-Thr-Gly (K-T-G). Aspartic acid (D), which precedes the triad, is usually conserved for this class, also. All the three motifs presented in this logo were expected for class A indicating the HMM profile were in accordance with the main conserved regions. Similar results were obtained for the HMM profiles of class C, and D of β-lactamases as is presented in Figures S2 and S3.

**2.1. Supplementary Figures**


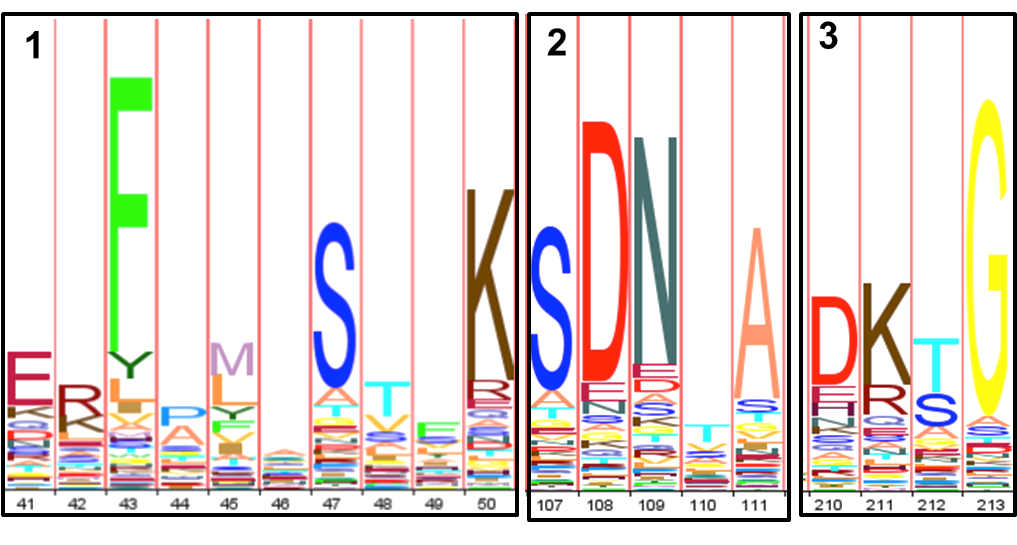


**Figure S1**. Illustrative image of the partial HMM profile logo containing the principal motifs of β-lactamase class A, generated by the LogoMat-M program, positioned on the boxes 1, 2 and 3. (1): First motif: the active site; (2): Second motif; (3) Third motif. The numbers placed below the capital letters indicate the amino acid position.


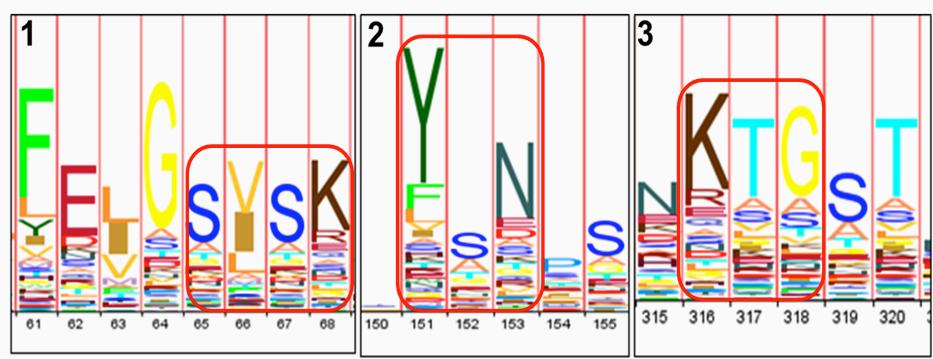


**Figure S2.** Illustrative image of the partial HMM profile logo containing the principal motifs of β-lactamase class C, generated by the LogoMat-M program, positioned on the boxes 1, 2 and 3. (1): First motif: the active site; (2): Second motif; (3) Third motif. The numbers placed below the capital letters indicate the amino acid position.


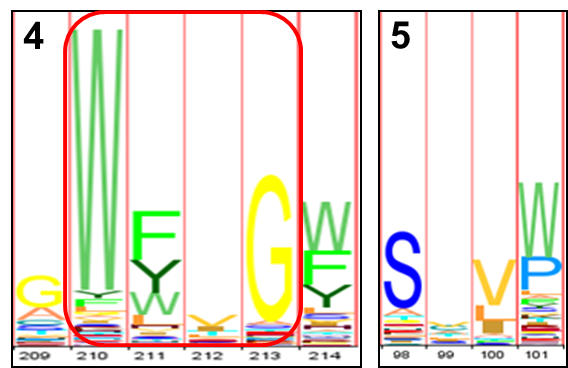

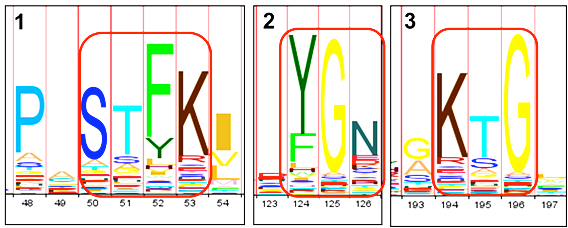


**Figure S3**. Illustrative image of the partial HMM profile logo containing the principal motifs of β-lactamase class D, generated by the LogoMat-M program, positioned on the boxes 1, 2 and 3. (1): First motif: the active site; (2): Second motif; (3) Third motif; (4) Fourth motif; (5) Fifth motif. The numbers placed below the capital letters indicate the amino acid position.


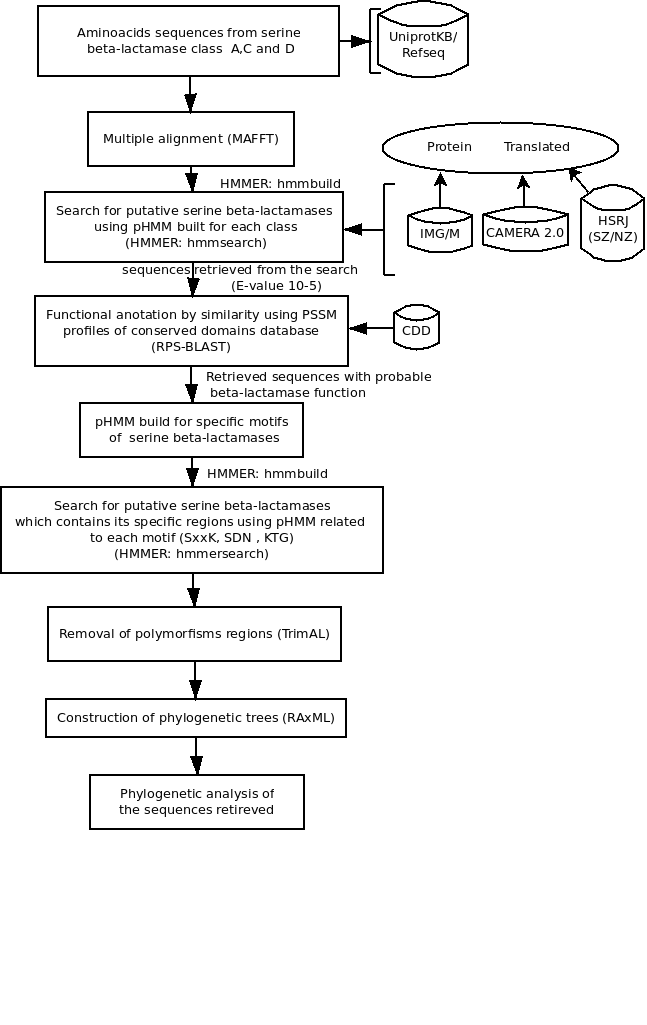


**Figure S4.** Schematic pipeline of the methodology involving the search for and diversity analysis of serine β-lactamases for each class (A, C and D), disposed on public databases and hospital sewages of South (SZ) and North Zone (NZ) of Rio de Janeiro (Brazil).

**Supplementary Table**

**Table S1.** Physical-chemical parameters of the hospital sewage samples from South Zone (SZ) and North Zone (NZ) from Rio de Janeiro (Brazil).

|  | **Samples** | |
| --- | --- | --- |
| **Parameters** | **South Zone (SZ)** | **North Zone (NZ)** |
| pH | 7,3 | 7,2 |
| DQO (mg /L) | 632 | 397 |
| DBO (mg /L) | 295 | 209 |
| P (mg /L) | 12,2 | 14,0 |
| N (mg /L) | 55,3 | 55,0 |
